# Viridans Group Streptococci Clinical Isolates: MALDI-TOF Mass Spectrometry versus Gene Sequence-Based Identification

**DOI:** 10.1371/journal.pone.0120502

**Published:** 2015-03-17

**Authors:** Silvia Angeletti, Giordano Dicuonzo, Alessandra Avola, Francesca Crea, Etleva Dedej, Francesca Vailati, Claudio Farina, Lucia De Florio

**Affiliations:** 1 Clinical Pathology and Microbiology Laboratory, University Hospital Campus Bio-Medico of Rome, Rome, Italy; 2 Microbiology Institute, AO ‘Papa Giovanni XXIII’ (formerly AO ‘Ospedali Riuniti’) of Bergamo, Bergamo, Italy; Queen Mary Hospital, the University of Hong Kong, HONG KONG

## Abstract

Viridans Group Streptococci (VGS) species-level identification is fundamental for patients management. Matrix-assisted laser desorption ionization—time of flight mass spectrometry (MALDI-TOF MS) has been used for VGS identification but discrimination within the Mitis group resulted difficult. In this study, VGS identifications with two MALDI-TOF instruments, the Biotyper (Bruker) and the VITEK MS (bioMérieux) have been compared to those derived from *tuf*, *soda* and *rpoB* genes sequencing. VGS isolates were clustered and a dendrogram constructed using the Biotyper 3.0 software (Bruker). *RpoB* gene sequencing resulted the most sensitive and specific molecular method for *S*. *pneumonia* identification and was used as reference method. The sensitivity and the specificity of the VITEK MS in *S*. *pneumonia* identification were 100%, while the Biotyper resulted less specific (92.4%). In non pneumococcal VGS strains, the group-level correlation between *rpoB* and the Biotyper was 100%, while the species-level correlation was 61% after database upgrading (than 37% before upgrading). The group-level correlation between *rpoB* and the VITEK MS was 100%, while the species-level correlation was 36% and increases at 69% if isolates identified as *S*. *mitis/S*. *oralis* are included. The less accurate performance of the VITEK MS in VGS identification within the Mitis group was due to the inability to discriminate between *S*. *mitis* and *S*. *oralis*. Conversely, the Biotyper, after the release of the upgraded database, was able to discriminate between the two species. In the dendrogram, VGS strains from the same group were grouped into the same cluster and had a good correspondence with the gene-based clustering reported by other authors, thus confirming the validity of the upgraded version of the database. Data from this study demonstrated that MALDI-TOF technique can represent a rapid and cost saving method for VGS identification even within the Mitis group but improvements of spectra database are still recommended.

## Introduction

The Viridans Group Streptococci (VGS) constitutes a heterogeneous group of bacteria. Most species are part of the normal flora in different tracts of human body, such as respiratory, urogenital and gastrointestinal. Despite their low virulence VGS have been associated with severe infections such as endocarditis, sepsis and abscesses in patients with different pathological conditions [1].

The very important human pathogen *S*. *pneumoniae* belongs to this group of bacteria causing community-acquired pneumonia, meningitis and otitis media. Clinical laboratories must be able to accurately differentiate *S*. *pneumoniae* from other VGS commonly found in clinical samples to facilitate appropriate antimicrobial therapy [2] but it is also important to identify the various species of VGS in some clinical settings, such as sepsis, endocarditis and in immunocompromised and transplant patients [1,3,4]. The taxonomy within this group of bacteria is not yet well defined and it is continuously updated by the results of the molecular typing methods [4–7].

Several nucleic acid—based technologies have been applied to the identification of VGS. Sequence analysis of the 16S rRNA gene has been widely applied to the taxonomy of streptococci, although the ability of this method to differentiate between closely related species, particularly *Streptococcus oralis* and *Streptococcus mitis*, has been debated [8]. Sequence amplification and analysis of several genes has been used for the species-level identification of streptococci such as *tuf* gene encoding elongation factor Tu, *rpoB* gene encoding RNA-polymerase beta-subunit and *sodA* gene encoding superoxide dismutase [7,9,10]. A variety of virulence and specific genes have been used as targets for a PCR-based discrimination of *S*. *pneumoniae* from other VGS, including genes encoding the virulence factors autolysin (*lytA*) and pneumolysin (*ply*) and the DNA fragment Spn9802 [2, 4,11].

However, most of these PCR assays are not able to discriminate *S*. *pneumoniae* from the recently discovered *Streptococcus pseudopneumoniae* and other streptococci belonging to the Mitis group.

Optochin sensitivity and bile solubility test are reliable test with adequate sensitivity and specificity, but optochin sensitivity needs a 18–24 hours incubation and bile solubility is subject to inter observer variability [12].

In recent years, the matrix-assisted laser desorption ionization-time of flight mass spectrometry (MALDI-TOF MS) technique has emerged as an alternative for clinical microbiology laboratories in the identification of a wide range of bacteria, mycobacteria, and fungi. The identification is based

on the protein composition of the microbial cell, particularly ribosomal proteins. The method is robust, inexpensive, and fast, and its advantages compared to conventional identification methods have been reported in several papers [13–14]. Commercially available MALDI-TOF MS systems provide reliable identification for many of clinically relevant bacterial species, and the underlying algorithms have been applied to viridans streptococci [15–16]. Nevertheless, the technique so far also failed at differentiating between *S*. *pneumoniae* and its closest relatives [10]. Several studies have been performed to increase the phylogenetic resolution of mass spectrometry by focusing the analysis on a predefined subset of discriminatory peaks [17]. In order to assess the performance of MALDI TOF MS method it is necessary to have a gold standard to which compare its results.

In the present study the potential MALDI-TOF MS technique in VGS identification was investigated applying two different MALDI-TOF instruments, MALDI-TOF biotyper (Bruker, Daltonics) and VITEK MS (bioMérieux, Marcy l’Etoile) to two sets of clinically relevant routine VGS isolates and comparing the results to those obtained by amplification and sequencing of *tuf*, *sodA* and *rpoB* genes.

## Methods

### Bacterial isolates

One hundred and fifty eight VGS strains, 70 isolated at the University Hospital Campus Bio-Medico of Rome, 85 at the AO ‘Papa Giovanni XXIII’ (formerly AO ‘Ospedali Riuniti’) and 3 ATCC strains (ATCC *S*. *pneumoniae* 49619, ATCC *S*. *thermophilus* 19258 and ATCC *S*. *equi* 43079) have been analyzed (S1 Table). The 155 VGS clinical isolates originated from 88 blood cultures, 52 respiratory samples, and 15 from other sites (vaginal samples, skin lesions, swabs…).

### Species identification

Bacterial identification was performed using the VITEK2 ID system (bioMérieux, Marcy l’Etoile, France), the MALDI-TOF (Microflex LT, Bruker Daltonics, Germany) with the MALDI Biotyper 3.0 software version, the MALDI-TOF VITEK MS-DS (bioMérieux, Marcy l’Etoile) and the sequence-based identification through *tuf*, *sodA* and *rpoB* gene sequencing.

### VITEK2 Compact identification

Clinical samples were inoculated on specific plates and incubated at 35°C in 5% CO_2_ to enable bacterial colonies to develop. After overnight incubation, the bacterial colonies that developed on the CNA agar medium were used to prepare a standardized inoculum, and the appropriate VITEK ID card. For identification of Gram-positive bacteria, the GP card (bioMérieux) was used, according to the manufacturer’s instructions. The VITEK-2 ID cards were logged and loaded into the VITEK-2 Compact system. The VITEK-2 Compact system automatically reported the results through software 05.01.

### Sequence-based identification

**S**equence-based identification for all isolates was achieved through *tuf*, *sodA* and *rpoB* gene sequencing and results obtained were compared to phenotypic tests (bile solubility and optochin susceptibility test), to *lytA* gene amplification by PCR and to MALDI-TOF identification.

Bacterial DNA was extracted by the EZ1 DNA tissue kit (Qiagen, Dusseldorf, Germany) following manufacturer’s instructions starting from 200 μL of bacterial pellet suspension. Five microliter of DNA was added to 45 μl of a PCR mixture containing 1X PCR Gold Buffer with MgCl2 (2.0 mM final concentration) (Applied Biosystems), 10mM deoxynucleotide triphosphate (dNTPs) (Applied Biosystems), 1.0 μM each of primers (Table 1) and 1.25 U of AmpliTaq Gold DNA polymerase (Applied Biosystems, Germany). PCR cycling parameters included an initial denaturation for 10 min at 94°C; 35 cycles of 1 min at 94°C, 1 min at 50°C, and 1 min at 72°C; and a final extension for 7 min at 72°C. Amplicons were purified with the Qiaquick PCR product purification kit (Qiagen, Germany) by following the manufacturer’s instructions.

**Table 1:** Sequences of oligonucleotides used in this study.

Gene	Primer Sequence 5’-3’	Reference
*tuf*	Str1 GTACAGTTGCTTCAGGACGTATC	[7]
	Str2 ACGTTCGATTTCATCACGTTG	
*rpoB*	rpoBF CCAAACGTYGGKGAAGATGC	[9]
	rpoBR TGIARTTTRTCATCAACCATGTG	
*sodA*	Sod1 CCITAYICITAYGAYGCIYTIGARCC	[18]
	Sod2 ARRTARTAIGCRTGYTCCCAIACRTC	
*lytA*	A750 GGCTACTGGTACGTACATTC	[19]
	A1145 AATCAAGCCATCTGGCTCTA	

*Tuf*, *sodA* and *rpoB* genes were sequenced using forward primers as in Table 1 [7,9,18] and the sequencing technology based on fluorescent dye terminator and ABI PRISM 3730 DNA Analyzer sequencer. The nucleotide sequences were compared to sequences present in the GenBank database using the BLAST programs (NCBI/BLAST, http://www.ncbi.nlm.nih.gov).

*LytA* PCR was performed as published by Messmer TO et al. using A750 and A1145 primers [19].

Comparing the tests on the basis of their sensitivity and specificity, a gold standard has been chosen.

### MALDI-TOF MS Biotyper identification (Bruker, Daltonics, Germany)

Bacterial colonies were grown overnight on sheep blood agar and subjected to ethanol-formic acid protein extraction according to the MALDI Biotyper protocol (Bruker Daltonics GmbH, Bremen, Germany) [20]. One microliter of the samples was prepared on a 96-spot polished steel target with 1μl of matrix solution (a saturated solution of α-cyano-4-hydroxycinnamic acid (Bruker, Daltonics GmbH, Bremen, Germany) in 50% acetonitrile and 2.5% trifluoroacetic acid (Sigma-Aldrich, Milano, Italy). MALDI-TOF MS was performed on a Microflex LT controlled by FlexControl version 3.4 software (Bruker). Spectra were acquired by the standard recommended proprietary method utilizing the Biotyper preprocessing standard method and the Biotyper Main-Spectrum (MSP) identification standard method (2,000 to 20,000 Da; linear positive method; laser frequency of 60 Hz). Species were identified using the MALDI Biotyper 3.1 (Bruker) and its standard database (Bruker Taxonomy database version 3.3.1). The software employed, Bruker Biotyper 3.1 (Bruker Daltonics GmbH, Bremen, Germany), automatically acquired spectra with fuzzy control of the laser intensity and analyzed them by standard pattern matching against the spectra of 5627 species used as reference data. After comparing the unknown spectra with all reference spectra in the database, the log scores were ranked. Values of >1.9 were required for secure identification at the species level, and values between 1.9 and 1.7 were required for secure identification at the genus level.

### Clustering of MALDI-TOF Spectra

Each isolate was loaded on ClinproTools by spectra grouping function, in order to allow to the software to group all the technical replicates in one biological replicate, named Class by software. A Class dendrogram of all the study isolates was constructed using the ClinproTool dendrogram creation standard method (with the correlation distance measured by the average linkage algorithm) of the Biotyper 3.1 software (Bruker Daltonics, Germany). Clusters were then detailed and analyzed according to arbitrary distance levels at 500, 180, 140 and 70.

### MALDI-TOF VITEK MS v2.0 identification (bioMérieux, Marcy l’Etoile)

Isolated bacterial colonies after ethanol-formic acid protein extraction were applied to a single well of a disposable, barcode-labeled target slide (VITEK MS-DS; bioMérieux, Marcy l’Etoile) using a 1.0-μl loop, overlaid with 1.0 μl of a saturated solution of alpha-cyano-4-hydroxycinnamic acid matrix in 50% acetonitrile and 2.5% trifluoroacetic acid (VITEK MSCHCA; bioMérieux, Marcy l’Etoile), and air dried. For instrument calibration, an *Escherichia coli* reference strain (ATCC 8739) was transferred to designated wells on the target slide using the procedure described above. The VITEK MS system includes an OEM (original equipment manufacturer)-labeled Shimadzu AXIMA Assurance mass spectrometer linked to a reference database, referred to as Knowledge Base. During target interrogation, mass spectra within a range of 2,000 to 20,000 Da are recorded in linear positive mode at a laser frequency of 50 Hz. For each interrogation, laser shots at different positions within the target well produces up to 100 mass profiles that are summed into a single, raw mass spectrum. The processed (binned) data are used to query Knowledge Base to determine the unknown’s taxonomic identity.

### Optochin susceptibility test for *S*. *pneumoniae* identification

A 5 μg optochin disk (6-mm disks; Liofilchem, Italy) was placed on sheep blood Columbia agar plates inoculated under 5% CO_2_ atmosphere. Optochin susceptibility was defined as an inhibition zone ≥14 mm.

### Bile solubility test for *S*. *pneumoniae* identification

The bile solubility test was performed with the tube method, with preparation of bacterial suspensions in 1 ml of 0.9% NaCl equivalent to a McFarland 1.0 standard. A 0.5-ml portion of 2% deoxycholate was added to a 0.5-ml suspension of each isolate prepared in 0.9% NaCl and incubated at 35°C for 1 h. A positive test was indicated by visible clearing of the suspension. A negative control for each isolate was similarly performed with 0.5 ml of 0.9% NaCl added to a 0.5 ml suspension of each isolate prepared in 0.9% NaCl.

## Results

A total of 158 VGS isolates of which 26 *S*. *pneumoniae* and 132 non pneumococcal VGS has been analyzed. 73/158 (46%) VGS strains of which 3 identified as *S*. *pneumoniae* and 3 ATCC strains (ATCC 49619, 19258 and 43079) have been analyzed at the University Hospital Campus Bio-Medico of Rome and 85/158 (54%) of which 22 identified as *S*. *pneumoniae* at the AO ‘Papa Giovanni XXIII’ (formerly AO ‘Ospedali Riuniti’) of Bergamo.

### *S*. *pneumoniae* identification

The sensitivity of the VITEK MS system for the identification of the 26 *S*. *pneumoniae* (25 clinical isolates and 1 ATCC 49619) was 100%, according to optochin susceptibility test, bile solubility test and *lytA* gene PCR. The specificity of the VITEK MS system for *S*. *pneumoniae* identification was 100% because the 132 non pneumococcal isolates (130 clinical isolates and 2 ATCC: *S*. *thermophilus* 19258 and *S*. *equi* 43079) were never identified as *S*. *pneumoniae* (Table 2).

**Table 2:** VGS isolates phenotypic and identification results.

Isolates	N°	Opt	BS test	*lytA*	VITEK Compact	MALDI-TOF Bruker	VITEK-MS	*tuf*	*sodA*	*rpoB*
					N°(%)	N°(%)	N°(%)	N°(%)	N°(%)	N°(%)
***S*. *pneumoniae***	26	S	Soluble	+	23 (88.5)	26 (100)	26 (100)	0 (0)	26 (100)	26 (100)
**Non pneumococcal viridans**	132	R	Not soluble	-	132 (100)	122 (92.4)	132 (100)	128 (97)	111 (84)	132 (100)

The sensitivity of the MALDI-TOF Biotyper system for the identification of the 26 *S*. *pneumoniae isolates* was 100%, according to optochin susceptibility tests, bile solubility test and *lytA* gene PCR. The specificity of the MALDI-TOF Biotyper system for *S*. *pneumoniae* identification was 92.4% (10 non pneumococcal isolates were misidentified as *S*. *pneumoniae*) after the last upgrade of the database (January 2014) and 49% with the precedent version (67 non pneumococcal isolates were misidentified as *S*. *pneumoniae*) (Table 2).

The VITEK Compact system correctly identified 23/26 (88.5%) *S*. *pneumoniae* isolates, these three misidentified *S*. *pneumoniae* were in two cases identified as *S*.*pneumoniae/S*. *mits* at the same score and in one case as *S*. *gordonii*. The specificity of the VITEK Compact system for *S*. *pneumoniae* identification was 100%, the 132 non pneumococcal isolates were never identified as *S*. *pneumoniae* (Table 2).

Current phenotypic identification procedures and MALDI-TOF systems were compared to sequence-based identification of three bacterial genes *tuf*, *sodA* and *rpoB* to achieve definitive identification in case of misidentification and to exceed the limited ability of these procedures to differentiate the *S*. *mitis* group, as well documented [18,21].

*RpoB* gene sequencing was the best sequencing method for pneumococcal as well as for viridans non pneumococcal streptococci identification, as reported in Table 2. In fact, *rpoB* identified *S*. *pneumoniae* and non-pneumococcal viridans streptococci concordantly with optochin, bile solubility and *lytA* amplification than *tuf* gene sequencing that never identified *S*. *pneumoniae* as a single match, but gave always at least two choices at the same score than *sodA* gene sequencing that identified *S*. *pneumoniae* strains with a specificity of 100% but in 21 cases misidentified non pneumococcal streptococci as *S*. *pneumoniae*.

### Nonpneumococcal identification performances

On the basis of MALDI-TOF identification and genes sequencing results it was possible to classify each isolates in six different groups as in Table 3.

**Table 3:** Non pneumococcal VGS isolates MALDI-TOF, VITEK II, *soda and tuf* identification versus *rpoB* genes sequencing: group concordance.

	Mitis group	Anginosus group	Bovis/Equinus complex	Salivarius group	Pyogenic group	Mutans group	Other	Correct ID
N°	95	15	13	5	2	1	1	132
**MALDI-TOF (Bruker)**	95/95 (100%)	15/15 (100%)	13/13 (100%)	5/5 (100%)	2/2 (100%)	1/1 (100%)	1/1 (100%)	132/132 (100%)
**MALDI-TOF (Bruker Last version)**	95/95 (100%)	15/15 (100%)	13/13 (100%)	5/5 (100%)	2/2 (100%)	1/1 (100%)	1/1 (100%)	132/132 (100%)
**MALDI-TOF MS (bioMérieux)**	95/95 (100%)	15/15 (100%)	13/13 (100%)	5/5 (100%)	2/2 (100%)	1/1 (100%)	1/1 (100%)	132/132 (100%)
***tuf***	94/95 (99%)	15/15 (100%)	12/13 (92%)	4/5 (80%)	2/2 (100%)	1/1 (100%)	1/1 (100%)	129/132 (98%)
***sodA***	95/95 (100%)	15/15 (100%)	9/13 (69%)	5/5 (100%)	2/2 (100%)	1/1 (100%)	1/1 (100%)	128/132 (97%)
**VITEK II**	88/95 (92.6%)	6/15 (40%)	12/13 (92%)	5/5 (100%)	2/2 (100%)	1/1 (100%)	1/1 (100%)	115/132 (87%)

The *tuf* gene misidentified species within the Bovis/Equinus group especially in two cases: *S*. *pasteurianus* and *S*. *thermophilus* were misidentified as strains belonging to the Mitis group than *rpoB* and *sodA* that correctly identified these isolates. Furthermore, *sodA* gene did not identify *S*. *lutetiensis*, correctly identified by *rpoB* and *tuf* genes.

Overall these data indicate that *rpoB* sequencing is the best genes sequencing method at the genus level for the identification of viridans streptococci including *S*. *pneumoniae* so that it should be chosen as the gold standard.

Using *rpoB* as gold standard, VGS were identified as follows: 26 *S*. *pneumoniae*, 95 non pneumococcal VGS belonging to the Mitis group, 15 to the Anginosus group, 13 to the Bovis group, 5 to the Salivarius group, 1 to the Pyogenic group, 1 to the Mutans group and 1 identified as *Abiotrophia*.

*RpoB* gene sequence-based VGS identification was compared to that obtained with VITEK II Compact, MALDI-TOF MS systems and the other two genes *sodA* and *tuf* and the correlation both at the group and at the species level were reported, as in Table 3 and Table 4.

**Table 4:** MALDI-TOF, *sodA* and *tuf* identification performance versus *rpoB* genes sequencing: ability to identify non pneumococcal VGS as single match (SM) or multiple match (MM).

	Species	MALDI-TOF (Bruker Last version)			MALDI-TOF MS (bioMérieux)			*Sod A* sequencing			*Tuf* sequencing			*RpoB* sequencing		
		MM	SM	SM ID %	MM	SM	SM ID %	MM	SM	SM ID %	MM	SM	SM ID %	MM	SM	SM ID %
**Anginosus group**	*S*. *constellatus*	0/4	4/4	100	0/4	4/4	100	0/4	4/4	100	1/4	3/4	75	0/4	4/4	100
	*S*.*anginosus*	0/8	8/8	100	0/8	8/8	100	0/8	8/8	100	5/8	3/8	37	0/8	8/8	100
	*S*. *intermedius*	0/3	0/3	0	0/3	0/3	0	0/3	0/3	0	0/3	0/3	0	0/3	3/3	100
**Bovis/Equinus complex**	*S*.*gallolyticus* subsp. *gallolyticus*	0/6	1/6	16.7	0/6	6/6	100	0/6	6/6	100	0/6	6/6	100	0/6	6/6	100
	*S*.*gallolyticus* subsp. *pasteurianus*	0/3	3/3	100	0/3	3/3	100	0/3	3/3	100	0/3	3/3	100	0/3	3/3	100
	*S*.*lutetiensis*	0/4	4/4	100	0/4	4/4	100	0/4	0/4	0	0/4	4/4	100	0/4	4/4	100
**Mitis group**	*S*. *mitis*	10/23	13/23	56	23/23	0/23	0	0/23	13/23	56	17/23	5/23	22	0/23	23/23	100
	*S*. *pseudopneumoniae*	0/17	0/17	0	0/17	2/17	12	0/17	0/17	0	14/17	0/17	0	0/17	17/17	100
	*S*. *oralis*	0/22	22/22	100	22/22	0/22	0	0/22	22/22	100	8/22	13/22	59	0/22	22/22	100
	*S*. *australis*	0/4	1/4	25	0/4	0/4	0	0/4	4/4	100	0/4	1/4	25	0/4	4/4	100
	*S*. *oligofermentas*	0/7	0/7	0	0/7	0/7	0	0/7	0/7	0	3/7	0/7	0	0/7	7/7	100
	*S*. *cristatus*	0/3	0/3	0	0/3	0/3	0	0/3	0/3	0	0/3	0/3	0	0/3	3/3	100
	*S*. *gordonii*	0/3	3/3	100	0/3	3/3	100	0/3	3/3	100	2/3	0/3	0	0/3	3/3	100
	*S*. *infantis*	0/4	1/4	25	0/4	0/4	0	0/4	3/4	75	3/4	0/4	0	0/4	4/4	100
	*S*. *sanguinis*	0/4	4/4	100	0/4	4/4	100	0/4	4/4	100	3/4	1/4	25	0/4	4/4	100
	*S*. *parasanguinis*	1/8	7/8	87	0/8	8/8	100		8/8	100	0/8	8/8	100	0/8	8/8	100
**Mutans group**	*S*. *mutans*	0/1	1/1	100	0/1	1/1	100	0/1	1/1	100	0/1	1/1	100	0/1	1/1	100
**Pyogenic group**	*S*.*dysgalactiae*	0/1	1/1	100	0/1	1/1	100	0/1	0/1	0	0/1	1/1	100	0/1	1/1	100
	*S*. *equi*	0/1	1/1	100	0/1	1/1	100	0/1	1/1	100	0/1	1/1	100	0/1	1/1	100
**Salivarius group**	*S*. *salivarius*	0/1	1/1	100	0**/**1	1**/**1	100	0/1	1/1	100	0/1	1/1	100	0/1	1/1	100
	*S*. *vestibularis*	0/2	0/2	0	0/2	0/2	0	0/2	2/2	100	0/2	2/2	100	0/2	2/2	100
	*S*.*thermophilus*	0/2	1/2	50	0/2	1/2	100	0/2	1/2	50	0/2	0/2	0	0/2	2/2	100
**Other**	*A*.*defectiva*	0/1	1/1	100	0/1	1/1	100	0/1	0/1	0	0/1	1/1	100	0/1	1/1	100

The group-level correlation between the two MALDI-TOF systems (Biotyper MS, Bruker and MALDI-TOF MS, Bio-Merieux) and *rpoB* was 100% (Table 3). The group-level correlation between the VITEK II Compact and the *rpoB* gene was 87% (Table 3). The group-level correlation between *tuf* and *sodA* versus *rpoB* genes was 98% and 97%, respectively (Table 3).

In Table 4, the species-level identification achieved by the two MALDI-TOF methods and *rpoB*, *sodA* and *tuf* genes sequencing is reported. *rpoB* gene sequencing is able to identify the larger number of VGS species; it is the only one to identify *S*. *intermedius* and *S*. *cristatus*.

The performance of the two MALDI-TOF methods is rather comparable, except for some species belonging to the Bovis/Equinus Complex and to the Mitis groups. In the Bovis/Equinus Complex, the MALDI-TOF Vitek MS correctly identifies *S*. *gallolyticus* subsp. *gallolyticus* in all cases, while the MALDI-TOF Biotyper misidentifies it as S. *gallolyticus* subsp. *pasteurianus*. In the Mitis group, *S*. *mitis* and *S*. *oralis* species are identified as single species only by the MALDI-TOF Biotyper in 13/23 (56%) and 22/22 (100%) cases respectively, while the Vitek MS always gives two possibility at the same score (*S*. *mitis*/*S*. *oralis*). In the Mitis group, the MALDI-TOF Biotyper never identified *S*. *pseudopneumoniae*, *S*. *oligofermentas* and *S*. *cristatus*, mainly identified as *S*. *mitis* or *S*. *oralis*, while the identification power was poor in case of *S*. *australis* and *S*. *infantis*, identified in a very low number of cases and mainly misidentified as *S*. *mitis*/*S*.*oralis* (Table 4).

Species-level identification was not possible with the MALDI-TOF VITEK MS for *S*. *australis* identified mainly as *S*. *mitis/oralis* or *S*. *parasanguinis*, for *S*. *oligofermentans S*. *cristatus* and *S*. *infantis* that were identified mainly as *S*.*mitis/S*.*oralis*. Within the Anginosus Group, 3 strains identified by the *rpoB* gene sequencing as *S*. *intermedius* resulted *S*. *anginosus* both with the MALDI-TOF Biotyper and with the VITEK MS. The databases of the two instruments contain a very low number of spectra for *S*. *intermedius*. In the Salivarius group, the two MALDI-TOF are not able to identify *S*. *vestibularis*; this species is represented in the database even if with a low number of spectra.

The species-level identification as single match was achieved by the two MALDI-TOF methods in 100% of cases for the following species: *S*. *mutans*, *S*. *dysagalactiae*, *S*. *equi*, *S*. *constellatus*, *S*. *anginosus*, *S*. *gallolyticus* subsp. *pasteurianus*, *S*.*lutetiensis*, *S*. *gordonii*, *S*. *sanguinis*, *S*. *salivarius*, *S*. *mutans* and *A*. *defectiva* (Table 4).

*Tuf* gene sequencing showed a very much lower accuracy than *rpoB* and *sodA* genes sequencing in the species-level identification of VGS, especially those belonging to the Mitis group; *tuf* gene identified the lower number of species and mainly as double match at the same score (Table 4). However, even if *sodA is* comparable to *rpoB* in the single match identification it exhibits less efficiency than *rpoB* gene in the number of species identified within the Mitis group (Table 4).

### Analysis of VGS MSP Dendrogram

The MSP dendrogram revealed three distinct clusters (I, II and III) according to an arbitrary cut-off at the distance level of 500.

**Fig 1:**
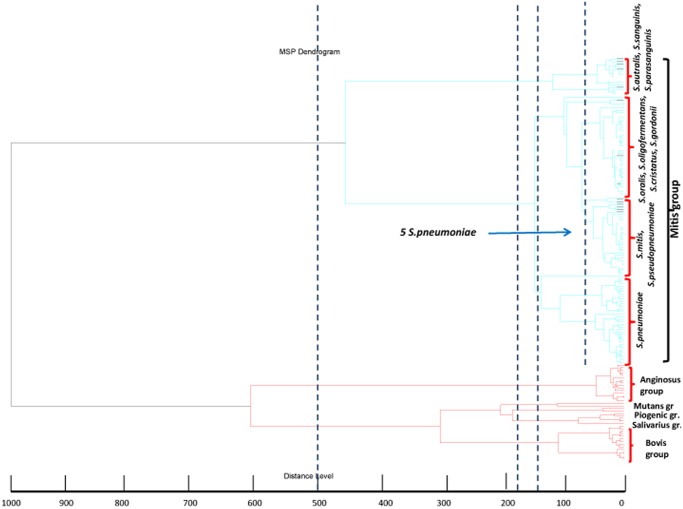
MSP classification dendrogram. Dotted lines position indicates the arbitrary distance levels at 500, 180, 140 and 70 used for strains clustering analysis.

Strains of Cluster I belonged to Bovis, Salivarius, Pyogenic and Mutans Group, strains in the Cluster II belonged to the Anginosus Group and strains in the Cluster III to the Mitis Group. Clustering the strains according to the arbitrary distance level of 180 and 140 significantly clustered the strains from the same group into the same cluster. At the distance level of 180, 7 clusters were found: Cluster I (Bovis Group), Cluster II (Salivarius Group); Cluster III (Pyogenic Group), Cluster IV (Mutans Group and Abiotrophia), Cluster V (Anginosus Group), Cluster VI (Mitis Group) and Cluster VII (*S*. *australis*, *S*. *parasanguinis*, *S*. *sanguinis* of the Mitis group). At the distance level of 140, 8 clusters were identified (Fig. 1): Cluster I (Bovis Group), Cluster II (Salivarius Group); Cluster III (Pyogenic Group), Cluster IV (Mutans Group and Abiotrophia), Cluster V (Anginosus Group), Cluster VI (*S*. *pneumoniae* strains), Cluster VII (Non pneumococcal VGS of the mitis group), Cluster VIII (*S*. *australis*, *S*. *parasanguinis*, *S*. *sanguinis*). To further analyze strains belonging to the non-pneumococcal VGS in the Mitis group we had to extend the analysis at the distance level of 70; in this way the Cluster VII was divided into two sub-clusters VIIa and VIIb mainly consisting of *S*. *mitis* or *S*. *pseudopneumoniae* strains (VIIa) and *S*. *oralis*, *S*.*oligofermentas*, *S*. *cristatus* or *S*. *gordonii* (VIIb) (Fig. 1). Effectively only 1/22 strains identified as *S*.*oralis* and 1/7 *S*. *oligofermentans* were grouped in the cluster VIIa, while 3/23 *S*. *mitis* and 1/ *S*. *pseudopneumoniae* were clustered in the cluster VIIb. All strains identified as *S*. *cristatus* and *S*. *gordonii* were clustered in the cluster VIIb as in the dendrogram.

## Discussion

Identification of VGS at the species level is an important factor influencing the clinical management of patients with VGS infection. Recently, several clinical diseases have been correlated to *S*. *mitis* infections and higher rates of *S*. *mitis* resistant to penicillin and fluoroquinolones have been described [22,23]. Furthermore, other VGS species such as *S*. *sanguini*s and *S*. *anginosus* have been correlated to endocarditis and gastrointestinal infections, respectively [23,24].

In the present study, the performance of two different MALDI-TOF systems in VGS identification was evaluated using as gold standard the *rpoB* gene sequencing giving the best performance in the identification of *S*. *pneumoniae* and of isolates belonging to the Bovis group than *sodA* and *tuf* genes sequencing (Table 3). Furthermore, *rpoB* gene sequencing identified the larger number of VGS species and always as single match than *sodA* and *tuf* genes. These last and particularly the *tuf* gene identify VGS species with multiple choices at the same score or no identified them, as also other authors have reported [4,21,25,26].

VGS strains identification from MALDI-TOF Biotyper and VITEK MS systems were 100% concordant at the group level with *rpoB* gene sequencing, while at the species level the concordance was lower especially in the Mitis group, as shown in Table 3 and 4. The MALDI-TOF Biotyper, after the release of its upgraded database (December 2013), gave best results in the identification of isolates belonging to the Mitis Group than VITEK MS, even if 10 isolates of the Mitis group were misidentified as *S*. *pneumoniae* while the VITEK MS resulted more accurate and precise and never gave false positive results in non-pneumocaccocal isolates (Table 2). For this reason strains identified with the Biotyper were further analyzed by the construction of a classification dendrogram based on spectra similarity through the use of the Biotyper 3.1 software (Fig. 1). MSP dendrogram analysis revealed that strains belonging to the different VGS groups are clustered separately, thus confirming the concordance found at the group level between MALDI-TOF systems and *rpoB* gene sequencing. In the Mitis group, 21/26 (91%) *S*. *pneumoniae* strains are clustered separately from non-pneumococcal VGS while 5/26, true *S*. *pneumoniae* strains, fall into the cluster of non-pneumococcal VGS of the Mitis group (arrow in Fig. 1) confirming in general the good performance of the MALDI-TOF in *S*. *pneumoniae* identification but indicating that despite the release of the upgraded database *S*. *mitis* may be still misidentified as *S*. *pneumoniae*.

Concerning non pneumococcal VGS in the Mitis group, two different clusters have been defined in the dendrogram at the distance level of 70: one cluster mainly consisting of *S*. *mitis* and *S*. *pseudopneumoniae* strains, thus confirming the difficulty of MALDI—TOF Biotyper to discriminate between the two species, and the second cluster mainly consisting of *S*. *oralis*, *S*. *oligofermentans*, *S*. *cristatus* and *S*. *gordonii*. This confirm the ability of the MALDI-TOF Biotyper to discriminate between *S*.*mitis* and *S*. *oralis*, identifying strains as single species than VITEK MS always giving a double species results (*S*.*mitis*/*S*.*oralis*), as reported in Table 4.

MALDI-TOF species-level identification was comparable for the two MALDI-TOF systems except for isolates in the Bovis and in the Mitis groups. In the Bovis group, the MALDI-TOF Biotyper was less accurate than VITEK MS in the identification of S. *gallolyticus* subsp. *gallolyticus* species because its database contains only one spectra for this species. The performance of the MALDI-TOF VITEK MS in VGS identification at the single species level was 100% for *S*. *pneumoniae* but very much lower for non-pneumococcal VGS in the Mitis group. This less accurate performance was due to the inability to discriminate between *S*. *mitis* and *S*. *oralis*. Strains identified with the *rpoB* gene sequencing as *S*. *mitis*, *S*. *oralis*, *S*. *pseudopneumoniae*, *S*. *australis*, *S*. *oligofermentans* and *S*. *cristatus* resulted always *S*. *mitis/S*.*oralis* at the VITEK MS. Noteworthy, *S*. *oligofermentas* and *S*. *australis* are not represented in the VITEK MS database, while *S*. *cristatus* is present but probably the number of spectra is too low to obtain a good performance of identification for these species. Conversely, the MALDI-TOF Biotyper (Bruker, Daltonics), after the release of the upgraded database, is able to discriminate between *S*. *mitis* and *S*. *oralis* giving always a single species identification in agreement with the *rpoB* gene sequencing. Species-level identification was not possible with the MALDI-TOF Biotyper for *S*. *oligofermentans* identified mainly as *S*. *oralis* because it is not represented in the database. Furthermore, the identification with the MALDI-TOF Biotyper of *S*. *cristatus* and *S*. *australis* was not achieved even if the database contains two and one spectra respectively; probably the number of spectra is too low to obtain a good performance of identification for these species and should be upgraded. Within the Anginosus Group 3 strains identified by the *rpoB* gene sequencing as *S*. *intermedius* resulted *S*. *anginosus* both with the MALDI-TOF Biotyper and with the VITEK MS. The databases of the two instruments contains *S*. *intermedius* spectra but probably the number of spectra are not sufficient to give the exact identification for this specie and should be upgraded. Species-level identification percentages achieved by the upgraded version are reported in Table 4. By this improvement the identification of non-pneumococcal VGS within the Mitis group was comparable to that achieved by molecular methods, such as *rpoB* gene sequencing.

In a recent paper, the complete genome sequences of 67 streptococci by genomic analyses such as multilocus sequence analysis (MLSA), average amino acid identity (AAI), genomic signatures, genome-to-genome distances (GGD) and codon usage bias has been performed. The authors defined MLSA, AAI and GGD robust markers to identify streptococci at the species level [27]. We compared the dendrogram obtained with the MALDI-TOF Biotyper (Fig. 1) to the phylogenetic trees constructed on the basis of MLSA and 16S rRNA analysis and we observed a good correspondence between the phylogenetic tree produced by genomic analysis and that from ribosomal protein spectra with the MALDI-TOF even if based on different methodologies. This data further confirm the robust clustering performed by the MALDI-TOF Biotyper on the basis of the spectra represented in its database.

In conclusion, MALDI-TOF technique can represent a reliable, rapid and cost saving method for VGS identification even within the Mitis group, but further improvements of VGS spectra database seem necessary.

## Supporting Information

S1 Table Phenotypic, molecular and MALDI-TOF characterization of VGS strains used in the study. (XLS)
